# Blind identification of state transitions and latent neural dynamics from electrophysiological recordings

**DOI:** 10.1016/j.jneumeth.2025.110600

**Published:** 2025-10-30

**Authors:** Addison L. Schwamb, Zongxi Yu, ShiNung Ching

**Affiliations:** Electrical and Systems Engineering, Washington University in St. Louis, St. Louis, 63130, MO, USA

**Keywords:** Neural modeling, Nonstationary dynamics, Latent state transitions

## Abstract

**Background:**

Neural dynamics change over time and with physiologic state. Modeling of neural dynamics can thus be understood at two levels: (i) identifying the latent process that governs how and when states change, and (ii) identifying the generative circuit mechanisms within each state.

**New method:**

Here, we develop a data-driven modeling method that tackles these two levels simultaneously. We formulate a parametric network model of neural dynamics that embeds state-dependent modulation. The modulation itself is controlled by a latent switching process, modeled as a Hidden Markov Model (HMM). A key challenge is that the model itself has internal states that must be estimated from observed data. This leads to a triune optimization problem, consisting of model parameterization of the HMM and neural dynamics, alongside state estimation. Our method brings together several optimization frameworks alongside estimation-theoretic constructs to solve this problem efficiently, enabling blind identification of state transitions and neural dynamics.

**Results:**

We validate this methodology on ground-truth data with known parameters, and find that it accurately infers the transitions in latent state and the dynamics of each state. We demonstrate its capability of inferring changes in brain dynamics from electrophysiological data by testing it on electroencephalography recordings with labeled state transitions.

**Comparison with existing methods:**

While similar methods exist to infer switches and dynamics on the level of individual neurons, there is no directly comparable method available for mesoscale modeling of neural circuits.

**Conclusions:**

Our methodology enables blind modeling of changing neural dynamics allowing for inference of modulatory circuit mechanisms.

## 1. Introduction

Elucidating how neural circuits reconfigure across internally and externally driven changes in state (or, dynamical regime) is an active problem in systems and computational neuroscience. States such as arousal, sleep, or task engagement are known modulators of circuit-level physiology, hence altering neuronal and synaptic parameters en route to changing circuit dynamics. These alterations, and their concomitant changes in neural computation, may be latent and difficult to directly observe.

The above challenge motivates the need for blind identification methods: methods that can infer both latent neural state transitions *and* the accompanying changes to neural parameters and dynamics, directly from recordings and without external labels. While prior work has addressed either latent state estimation (e.g., hidden Markov models or switching dynamical systems) or system identification (e.g., parametric dynamical systems modeling), there are fewer approaches that address these problems jointly. Here, we aim to address this challenge and, crucially, to do so in a manner that offers biological interpretability at the level of individual subjects. Specifically, we propose a data-driven modeling framework for the simultaneous blind identification of state changes and circuit dynamics at whole-brain scales. As in our recent developments (Schwamb et al., 2025), our approach is based on the premise that circuit changes arise from modulation of a baseline or ‘rest’ model parameterization. By coupling state segmentation with local identification of these modulatory changes, we seek to recover interpretable models of circuit dynamics *and* identify when and how these dynamics become modulated across time.

### 1.1. Parametric neural modeling at whole-brain scales

As indicated, data-driven modeling or system identification (i.e., ‘fitting’), of neural dynamics is itself an active area of research, including at whole-brain spatial scales (Breakspear, 2017; Schirner et al., 2015). Here, we restrict our attention to parametric dynamical systems models with biophysically formulated variables, where the goal of fitting is to find model parameters that minimize predictive errors or losses. In this research space, platforms such as the Human Neocortical Neurosolver (Neymotin et al., 2020) and the Virtual Brain (Sanzleon et al., 2013) enable the identification of model parameters for individuals based on their recordings of their data brain activity. Recently, we developed the mesoscale individualized neural dynamics (MINDy) modeling approach for capturing whole-brain dynamics at the mesoscale, i.e., neural population level (Singh et al., 2020, 2025), and the current work continues developing this framework in the context of nonstationary dynamics.

### 1.2. Nonstationarity and latent state identification

Indeed, the above methods are in most cases limited to a single state or regime, i.e., with a tacit assumption of stationarity in the data from which the models are fit. Brain data (and brain dynamics), however, are fundamentally *nonstationary*. Dynamics of brain data change temporally as subjects transition from rest to task (Grigg and Grady, 2010), sleep to wake (Nir and de Lecea, 2023), as well as with pathology (Manuca et al., 1998) or pharmacological interventions (Ching et al., 2010). For this reason, stationary modeling methods are not well suited for use with neural recordings that span long times scales or multiple states.

This gap has led to engagement of the problem of modeling non-stationary brain dynamics, especially in an individualized, data-driven manner. The most common approach is to layer a switching model on top of the neural dynamics. For instance, prior work has used switched linear dynamical systems (SLDS) to model brain data (Glaser et al., 2020; Hu et al., 2024). In these approaches, a latent variable dictates switches between discrete sets of linear dynamics (Song et al., 2022; Song and Shanechi, 2023; Weng et al., 2024). Often the dynamics of the latent variable itself are also modeled, e.g., by using a hidden Markov model (HMM) (Rabiner, 1989) to dictate the dynamics of the switches (He et al., 2023; Li et al., 2024). Other researchers have created more complex nonlinear models with switched recurrent neural networks (Karniol-Tambour et al., 2024; Zhang and Saxena, 2024).

**Fig. 1.**
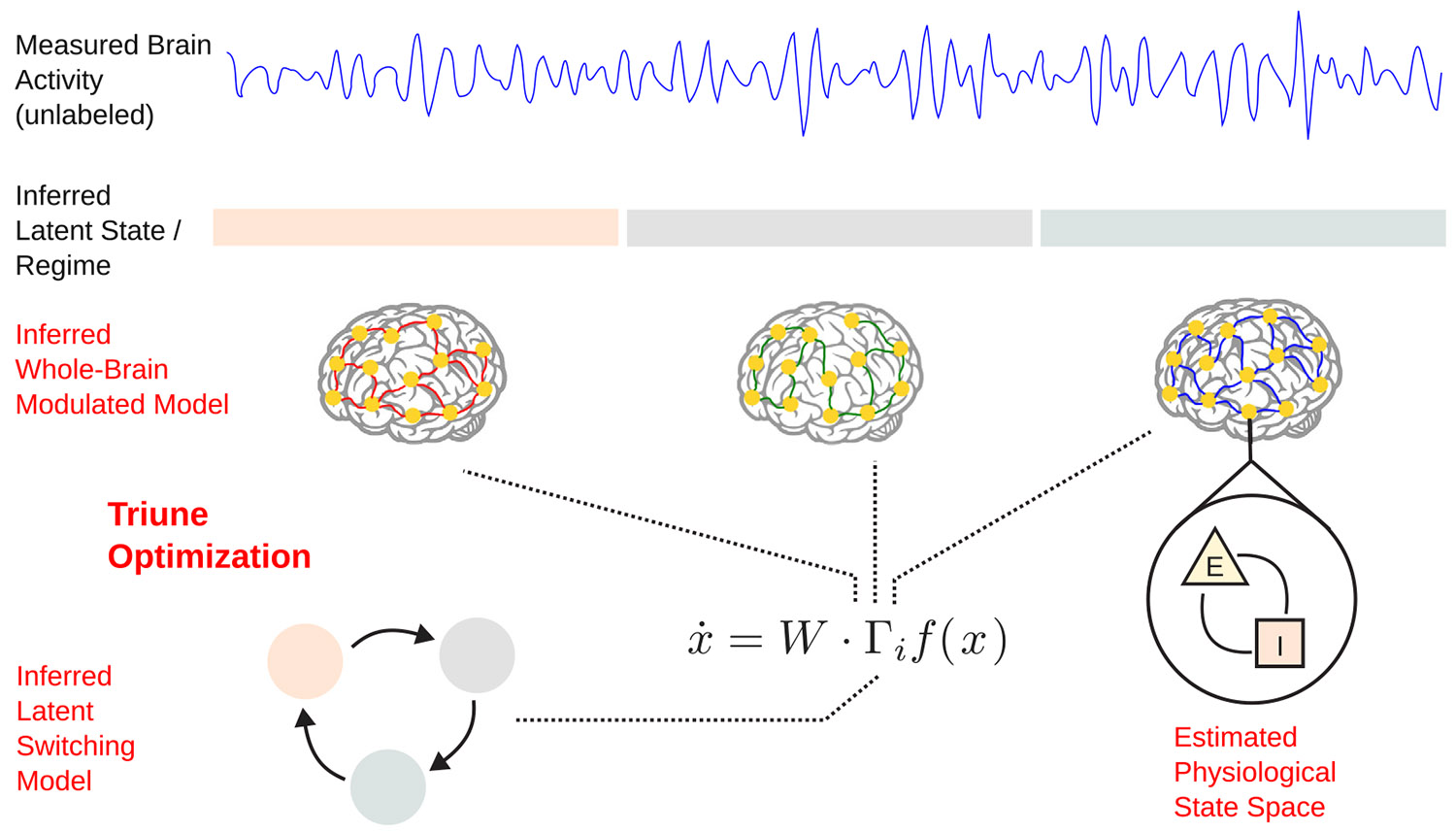
Approach to blind identification and model inference. In this paper we take unlabeled, nonstationary brain activity, and simultaneously make inferences at three levels: (i) the latent state/dynamical regimes of the data along with the dynamics describing their switches, (ii) the dynamics of a full-brain model of each latent state, and (iii) the latent physiological state space (i.e., the activation of each neural population).

Our approach in this paper is similar insofar as we combine a switching model with a neural dynamical model, but with three key distinctions. This approach is schematized in Fig. 1. First, we implement specific constraints on the parameters of our model, building from our prior MINDy framework, that enable mechanistic interpretations to be made based on the fit parameters. For example, we impose specific excitatory and inhibitory structures onto our connection weight matrix, enabling inference of excitatory/inhibitory circuit dynamics in the brain. This avoids the ‘black box’ problem of fitting an unconstrained RNN to neural data which may provide good predictions, but few mechanistic interpretations.

In this regard, we tailor our method to noninvasive recording modalities common in human studies — specifically in this paper, electroencephalography (EEG), though our method is amenable to functional neuroimaging and magnetoencephalography (MEG) as well. When working with EEG, we face the problem of neural populations which are not directly observed. Inhibitory neurons are theorized to be too distant from the surface of the cortex to directly contribute to EEG signals (Buzsáki et al., 2012), and so their contributions to brain dynamics must be indirectly inferred. This means that we have an *underdetermined* system, in which the dimension of the observed data (the number of EEG channels) is lower than the number of neural populations we would like to model. Current switched RNN models, e.g., Karniol-Tambour et al. (2024) and Zhang and Saxena (2024) use variational inference to construct a low-dimensional dynamics from the observed data. This works well when the goal is to directly infer low-dimensional dynamics from high-dimensional neural data, but degrades when used for making inferences in higher dimensional spaces (Agrawal et al., 2022; Wei and Sun, 2025), as we seek to do here. To address this problem, we use a version of the extended Kalman filter (EKF) (Kalman, 1960; Julier and Uhlmann, 1997), already validated in stationary M/EEG dynamical systems models (Singh et al., 2025; Schwamb et al., 2024). We therefore have a triune optimization problem: we must estimate (i) the transitions in latent state/dynamic regime, (ii) the whole-brain dynamical models of each dynamic regime, and (iii) the state space of these dynamical models, i.e., the activity of each neural population at any given time point.

Our final distinction from previous methods is the formulation and implementation of a modulation architecture in our model. Rather than having completely distinct recurrent weight matrices as in Karniol-Tambour et al. (2024) and Zhang and Saxena (2024), we use a base weight matrix that is constant across all dynamic regimes, which is modulated (multiplied element-wise) by a modulation matrix which switches with each change in dynamical regime. This enables a level of inference regarding the decomposition of changes into a persistent baseline and state-dependent changes. Such a decomposition is harder to obtain from a model with entirely distinct parameters for each regime. To the best of our knowledge, this formulation and overall goals are most aligned with the prior approach developed in Li et al. (2024), which also seeks to identify blind latent changes in neural data. Our approach is distinct in a few important ways, notably that we build dynamical systems models at whole brain scales, vs. statistical models at neuronal scales commensurate with spiking activity. Accompanying these scale distinctions are important methodological differences that we will later discuss.

Below, we develop and present the proposed methodology. Our model follows from of our prior data-driven modeling approaches MINDy (Singh et al., 2020, 2025) (which developed a framework for parametric whole-brain modeling of stationary data) and modulated MINDy (Schwamb et al., 2025) (which developed a framework for non-stationary modeling when latent state labels are known). Here, we add the additional critical capability of blind identification of different latent states/dynamical regimes. That is, we identify the (unknown) regimes alongside reconstruction of the mesoscale dynamics operative in each of the inferred regimes. We accomplish this by adding a HMM, which models the changes in dynamical regimes, and dictates the switching of the modulation matrix in a modulated MINDy architecture. We call this augmented modeling framework HM-MINDy, for Hidden Markov MINDy. We validate this on simulated data, to test the accuracy and reliability of our model in recovering known ground truth parameters. We then test our model on the ability to infer distinct neural mechanisms associated with alpha waves, on an open EEG dataset of resting eyes open and eyes closed recordings. These data allow us to further validate the reliability and individuality of our fitted models.

## 2. Methods

### 2.1. Model formation

Our model is adapted from our prior whole-brain dynamical systems framework in Singh et al. (2020, 2025), and especially the modulated architecture in Schwamb et al. (2025). The model equations for the modulated model are given below, with details provided in Appendix A.


(1) $$x_{i+1}-x_{t}=(W⊙\Gamma_{i})\tanh(\mathrm{Sx}+V)+\mathrm{Dx}+C+\epsilon .$$

(2) $$y_{t}={\mathrm{Hx}}_{t}+\nu$$

Here, $x_{t}\in R^{n}$ is the neural activity of n neural populations at time t, W represents the base neural connectivity weight matrix, and $\Gamma_{i},i\in \{1,2,\ldots ,m\}$ represents m distinct modulations of the connectivity. The diagonal matrix D parameterizes the self-decay (i.e., leak) of each population, S and V parameterize the slope and offset of the nonlinear activation function, and C is the bias of each population. The vector $y_{t}$ is the measured (EEG) signal at time t, and H is the lead-field matrix. Process and observation noise are captured in ϵ and ν, respectively. The modulation structure of this model is schematized in Fig. 1. To preserve the signed connectivity structure of W (see Appendix A), entries in each $\Gamma_{i}$ matrix are constrained to be positive or 0. This enables a modulation which scales entries in W by varying amounts, without affecting the directional structure of the connectivity.

#### 2.1.1. Hidden Markov model

These modulatory matrices are switched via a Hidden Markov Model (HMM) (Rabiner, 1989), which controls i, i.e., which Γ matrix is ‘active’ at any given point in time. An HMM is described by three parameters, typically denoted as $A\in R^{n\times n}$, $b\in R^{m\times n}$, and $\pi \in R^{n}$. A is the transition probability matrix, dictating the probability of transitioning from any one state to any other state:

(3) $$A=\left[\begin{array}{ccc} P(s_{t+1}=1|s_{t}=1) & \ldots & P(s_{t+1}=1|s_{t}=m)\\ \vdots & \ddots & \vdots \\ P(s_{t+1}=m|s_{t}=1) & \ldots & P(s_{t+1}=m|s_{t}=m) \end{array}\right]$$

In this way, A enables calculation of state probabilities over time via the simple relation

(4) $$P(s_{t+1})=AP(s_{t}).$$

The probability of the initial state is denoted by π:

(5) $$\pi =\left[\begin{array}{c} P(s_{0}=1)\\ \vdots \\ P(s_{0}=m) \end{array}\right],$$

and b is the observation probability of each state. In a system with discrete observations, b is formulated as a matrix. In our problem, we do not have discrete observations as our observations are the voltages of the EEG signal at each timepoint, which vary continuously. Therefore, we use a continuous measure of observation probability rather than a discrete matrix. Our choice of measure for observation probability is discussed in detail below. The other parameters describing an HMM, A and π, are inferred based on the data.

### 2.2. Model fitting procedure

#### 2.2.1. Triune estimation problem

Our goal, succinctly, is to identify the parameters of the HMM *and* the constituent MINDy models. However, within the latter exists what is known as a dual inference problem: (i) estimate $x_{t}$ and (ii) estimate the model parameters {W,Γ,D,S,V,C}, from the observable (i.e., EEG) data. This inference problem is due to the fact that inhibitory neurons are theorized to not directly contribute to the EEG signal (Buzsáki et al., 2012), and hence the lead field matrix H is not invertible. Thus, in total, we face a *triune estimation problem* involving neural states (x), neural model parameters (W, etc.) and HMM parameters and probabilities (A, etc.).

Papers solving similar problems (e.g., Karniol-Tambour et al. (2024) and Zhang and Saxena (2024)) have typically taken approaches such as variational inference, which are suited to dimensionality *reduction*. Inferring a MINDy model, however, is essentially a dimension expansion (going from EEG to underlying E–I circuit dynamics). To address this, we adopt the iterative estimation approach detailed in Singh et al. (2025) and Schwamb et al. (2024, 2025). This approach uses an Extended Kalman Filter (Kalman, 1960; Julier and Uhlmann, 1997) integrated with backpropagation of the parameter and noise covariance error gradients to simultaneously estimate the latent state $x_{t}$ and the parameters {W,Γ,D,C,S,V}. Full details of this approach, adapted from Singh et al. (2025) and Schwamb et al. (2024, 2025), are given in Appendix B.

#### 2.2.2. Fitting MINDy parameters

In the expanded modulated MINDy model used here, only one of the $\Gamma_{i}$ matrices is used at each timepoint based on the modulation labels. Since our current problem is blind identification of changes in modulation, we use a current estimate of the modulation label to select the appropriate $\Gamma_{i}$ for model evolution and gradient calculation.

To aid in the tractability of the problem, we implement a sparsity constraint on W and a rank constraint on Γ. We constrain W’s excitatory–excitatory ($W_{ee}$) and excitatory–inhibitory ($W_{ei}$) submatrices to have 75% of their non-diagonal connections be zero, randomly selected. This is motivated by previous work using structural connectivity data in conjunction with MEG data being modeled with (unmodulated) MINDy (Singh et al., 2025). We replicated our first experiment (Section 2.3.1) with no constraints on our $W_{ee}$ and $W_{ei}$ submatrices. In addition to constraining the excitatory–excitatory and excitatory–inhibitory submatrices of W, we also constrain the inhibitory–excitatory and inhibitory–inhibitory submatrices ($W_{ie}$ and $W_{ii}$ to be diagonal matrices. This decision is motivated by the understanding that inhibitory neurons have only local connections (Buzsáki et al., 2012). By making the IE and II submatrices diagonal, we ensure that inhibitory populations only have local connections. Finally, we constrain each $\Gamma_{i}$ to be rank 1, i.e., the outer product of two n-dimensional vectors. This assumption is motivated by the premise that neuromodulators act in a spatially diffuse manner (Marder and Thirumalai, 2002). Because each $\Gamma_{i}$ is multiplied element-wise by W, the excitatory submatrices of each $\Gamma_{i}$ also effectively have 75% of their elements set equal to 0, though we do not enforce this condition on Γ directly.

To fit the model parameters, we use NADAM (Dozat, 2016), implemented with PyTorch’s Autograd engines (Mazza and Pagani, 2021). This improves fitting efficiency and scalability relative to our prior implementations (Singh et al., 2020, 2025; Schwamb et al., 2024), by allowing GPU acceleration. Autograd combines both the local linearization and covariance update processes of the Kalman filter into the backward operation. Notably, this significantly reduces the number of iterations needed for the convergence of the backpropagated Kalman filter approach.

#### 2.2.3. Inferring HMM and modulation labels

In contrast to previous work (Schwamb et al., 2025), we want to be able to identify the modulation labels *blindly*, using the dynamics of the Hidden Markov Model. To accomplish this, we add an ‘outer loop’ to the Kalman filtering/parameter fitting steps described above, which handles the inference of the modulation labels, as well as the HMM parameters {A,π}. Our entire procedure is provided in Algorithm 1, below. To elaborate, we initialize the model with random modulation labels and run the ‘inner loop’ Kalman filtering and parameter gradient steps through the full length of the data one time. This allows the model parameters to be updated so that they are in the general vicinity of values which will fit the data.

We then Kalman filter the entire length of the data m times using each $\Gamma_{i}$, e.g., if m=2, we will Kalman filter the data using $\Gamma_{1}$ for all timepoints, then filter the data again using $\Gamma_{2}$ for all timepoints. Each time we do this, we save the *a posteriori* Kalman residuals ${\tilde{y}}_{k|k}$ for use as our observation likelihood. By using a continuous observation likelihood measure rather than a discrete observation matrix b, we can handle the continuous observations given by the EEG data. Since our base method is predicated on finding parameter values which enable good one-step Kalman prediction of the neural state (x), we also use Kalman residuals to determine which modulation matrix ($\Gamma_{i}$) is most likely to have produced the observed data. Since the lowest residuals indicate the best fitting parameter values (and therefore the most likely modulation matrix), we invert the residuals so that the lowest residuals give the highest observation likelihood.

Once we have the observation likelihoods given by the inverted Kalman residuals, we can then use conventional methods to infer the modulation states and the HMM parameters. The forward–backward algorithm (Rabiner, 1989) provides an estimate of the probability of each HMM state at each timepoint, enabling us to identify the most likely sequence of modulation states given the observation probabilities and the current HMM parameters. This estimated sequence of modulation states can then be used in the Baum-Welch algorithm (Rabiner, 1989) to update the HMM parameters A and π. The estimated sequence of modulation states can also be used as the modulation labels for another Kalman filtering/parameter fitting (‘inner loop’) iteration through the data.

On occasion, the outer loop state estimation algorithm can fall into pathological solutions involving only a single modulation state. This can happen when one of the $\Gamma_{i}$ matrices yields a lower inverted Kalman residual for all timepoints compared to the other $\Gamma_{i}$ matrices. When this happens, the variance of the estimated HMM state probabilities is quite low. To avoid situations such as this early in the optimization process, we enact a threshold hyperparameter on the variance of the estimated HMM state probabilities (δ=0.1), and if the variance is lower than the threshold, we K-means cluster the Kalman residuals smoothed with a local smoothness prior. This enables the inner loop to continue fitting all of the Γ matrices on different timepoints until multiple $\Gamma_{i}$ matrices are commensurate with the data and can be fine-tuned to capture dynamic changes that vary temporally. When the state estimation process yields HMM probabilities which are a higher variance than our threshold (i.e., there are transitions between two or more states), the K-means clustering step is not necessary.

**Table:** 

Algorithm 1 Hidden Markov Mesoscopic Individualized Neurodynamics (HM-MINDy)
$$\begin{array}{cc} \; & \;\mathrm{Require}:\;\text{Observations}\;\{y_{1},\ldots ,y_{T}\},\;\text{modulation matrices}\;\{\Gamma_{1},\ldots ,\Gamma_{m}\},\\ \; & \;\;\;K\text{-means threshold}\;\delta ,\;\text{entropy threshold}\;\varepsilon ,\;\text{maximum iterations}\\ \; & \;\;\;N_{\max}\\ \; & \;\mathrm{Ensure}:\;\text{Modulation labels}\;\{z_{1},\ldots ,z_{T}\},\;\text{HMM parameters}\;A,\pi ,\;\text{model}\\ \; & \;\;\;\text{parameters}\;W,\Gamma ,D,C,S,V,Q\\ \; & \;1:\;\text{Initialize}\;\;\text{HMM}\;\;\text{parameters}\;\;\{A,\pi \},\;\;\text{model}\;\;\text{parameters}\\ \; & \;\;\;\{W,\Gamma ,D,C,S,V,Q\}\\ \; & \;2:\;\text{Initialize modulation labels}\;z_{t}\;\text{by simulating HMM for all}\;t\\ \; & \;3:\;\mathrm{for}\;n=1\;\text{to}\;N_{\max}\;\mathrm{do}\\ \; & \;4:\;\;\;\text{Backpropagated Kalman filtering and parameter update using}\\ \; & \;\;\;\{z_{t}\}\\ \; & \;5:\;\;\;\mathrm{if}\;\text{not converged}\;(\text{i.e.},\;\text{mean Shannon entropy of}\;\gamma_{t}>\varepsilon )\;\mathrm{then}\\ \; & \;6:\;\;\;\;\;\mathrm{for}\;i=1\;\text{to}\;m\;\mathrm{do}\\ \; & \;7:\;\;\;\;\;\;\;\text{Run Kalman filter over entire sequence using}\;\Gamma_{i}\\ \; & \;8:\;\;\;\;\;\;\;\text{Compute and store residuals}\;{{\tilde{y}}_{t}^{\left(i\right)}\}}_{t=1}^{T}\\ \; & \;9:\;\;\;\;\;\mathrm{end}\;\mathrm{for}\\ \; & \;10:\;\;\;\;\;\mathrm{for}\;t=\;\text{to}\;T\;\mathrm{doo}\\ \; & \;11:\;\;\;\;\;\;\;\mathrm{for}\;t=1\;\text{to}\;T\;\mathrm{do}\\ \; & \;12:\;\;\;\;\;\;\;\;\;\text{Compute likelihood:}\ell_{t}^{\left(i\right)}\leftarrow \frac{1}{{\tilde{y}}_{t}^{\left(i\right)}}\\ \; & \;13:\;\;\;\;\;\;\;\mathrm{end}\;\mathrm{for}\\ \; & \;14:\;\;\;\;\;\mathrm{end}\;\mathrm{for}\\ \; & \;15:\;\;\;\;\;\text{Run Forward-Backward using}\;\{\ell_{t}^{\left(i\right)}\}\;\text{and}\;(A,\pi )\;\text{to get}\;\gamma_{t}(i)\\ \; & \;16:\;\;\;\;\;\text{Normalize}\;\gamma_{t}(i)\;\text{over}\;i\;\text{to get}\;p(z_{t}=i|y_{t})\\ \; & \;17:\;\;\;\;\;\mathrm{if}\;{\text{Var}}_{t}[\gamma_{t}(i)]<\delta \;\text{for all}\;i\;\mathrm{then}\\ \; & \;18:\;\;\;\;\;\;\;\text{Smooth residuals temporally}\\ \; & \;19:\;\;\;\;\;\;\;\text{Run K-means on smoothed residuals with}\;k=m\\ \; & \;20:\;\;\;\;\;\;\;\text{Set}\;z_{t}\;\text{to cluster assignments}\\ \; & \;21:\;\;\;\;\;\mathrm{else}\\ \; & \;22:\;\;\;\;\;\;\;\mathrm{for}\;t=1\;\text{to}\;T\;\mathrm{do}\\ \; & \;23:\;\;\;\;\;\;\;\;\;\text{Set}\;z_{t}\leftarrow \text{ar}\;\max_{i}\gamma_{t}(i)\\ \; & \;24:\;\;\;\;\;\;\;\mathrm{end}\;\mathrm{for}\\ \; & \;25:\;\;\;\;\;\mathrm{end}\;\mathrm{if}\\ \; & \;26:\;\;\;\;\;\text{Update}\;(A,\pi )\;\text{using Baum-Welch}\\ \; & \;27:\;\;\;\mathrm{end}\;\mathrm{if}\\ \; & \;28:\;\mathrm{end}\;\mathrm{for}\\ \; & \;29:\;\mathrm{return}\;\{z_{t}\},\;A,\pi ,\;\{W,\Gamma ,D,C,S,V,Q\} \end{array}$$

These estimation techniques (i.e., the ‘outer loop’ and the ‘inner loop’) alternate until the forward–backward algorithm finds a probability sequence which has sufficiently low mean Shannon entropy. A low Shannon entropy value for each timepoint indicates that there is high probability for one modulation state, and low probability for the others (Shannon, 1948) - i.e., the state is inferred with a high amount of certainty. At this point, the modulation labels are no longer re-estimated and the fitting continues in the inner loop only, to further tune the model parameter values. The threshold for low enough entropy is a hyperparameter which can be tuned based on the data and the number of modulation states m (a higher m leads to higher estimated entropy), but in our simulations we used a threshold of ε=0.15. Additionally, the variance of the HMM state probabilities must be higher than our threshold for reverting to K-means in order for state estimation to stop (i.e., we do not stop fitting on a solution found with K-means).

### 2.3. Simulation and actual data

#### 2.3.1. Synthetic data

To enable the validation of our fitting procedure, we created synthetic data with known ground-truth parameters, as we did in Schwamb et al. (2025). To create this synthetic data, we established models by combining fixed parameter values with values drawn from random distributions. The fixed values and distributions for the model parameters are listed in Table 1.

We constructed our W submatrices to reflect priors about the connectivity structure of the brain, namely that whole-brain connectivity typically manifests as a ‘backbone’ of connections (e.g., networks identified by functional connectivity methods) with a few additional connections (Singh et al., 2020). To reflect this, W submatrices were constructed as a linear combination of a low-rank matrix (representing the network ‘backbone’) and a sparse matrix (representing the sparse additional connections) via:

(6) $$W_{\text{ee}}=W_{s}=W_{11}W_{12}^{T}+W_{\text{diag}}.$$

where $W_{s}\in R^{n\times n}$ is a sparse matrix, $W_{11},W_{12}\in R^{n\times n∕4}$ are low rank matrices, and $W_{\text{diag}}\;\in \;R^{n}$ is a vector specifying the diagonal self-connection weights. Note that (6) specifies $W_{\text{ee}}$, but both $W_{\text{ee}}$ and $W_{\text{ei}}$ were constructed in this way. $W_{\text{ie}}$ and $W_{\text{ii}}$ were constructed as diagonal matrices with diagonal values directly sampled from their distributions.

We initialized each $\Gamma_{i}$ matrix as the outer product of a vector drawn from a unique distribution, representing a modulation that is similarly low-rank as the $W_{11}$ and $W_{12}$ components of the W submatrices. To construct each unique distribution, we first randomly generate its mean, $\mu_{i}$, from 𝒩(1,0.1). We then generate a random binary digit indicating whether we should use a uniform distribution, or a normal distribution. Then, we generate a variance $\sigma_{i}$ for the distribution. If we are using a uniform distribution, $\sigma_{i}∼𝒩(0.4,0.1)$, and if we are using a normal distribution, $\sigma_{i}∼𝒩(0.05,0.01)$. Then, we generate a vector, $\Gamma_{i,k}$, from the constructed distribution: if uniform, $\Gamma_{i,k}∼𝒰(\mu_{i}-(\sigma_{i}∕2),\mu_{i}+(\sigma_{i}∕2))$; if normal, $\Gamma_{i,k}∼𝒩(\mu_{i},\sigma_{i})$. Then, $\Gamma_{i}=\Gamma_{i,k}\Gamma_{i,k}^{T}$. To test sensitivity to different ground truth correlations between $\Gamma_{i}$ matrices, we used a multivariate normal distribution:

(7) $$\Gamma_{k}=𝒩(\mu ,\Sigma ),$$

where μ and Σ are constructed as follows:

(8) $$\mu =\left[\begin{array}{c} \mu_{1}\\ \vdots \\ \mu_{m} \end{array}\right],$$

(9) $$\Sigma =\left[\begin{array}{c} \sigma_{1}\\ \rho \\ \vdots \\ \rho \end{array}\;\begin{array}{ccc} \rho & \cdots & \rho \\ \sigma_{2} & \cdots & \rho \\ \vdots & \ddots & \vdots \\ \rho & \cdots & \sigma_{m} \end{array}\right].$$

Here, $\mu_{i}$ represents the mean of each $\Gamma_{i,k}$ vector, $\sigma_{i}$ represents the variance, and ρ represents the cross-covariance between $\Gamma_{i,k}$ vectors. The distributions for each $\Gamma_{i,k}∼𝒩(\mu_{i},\sigma_{i})$ vary slightly in mean and variance, with $\mu_{i}∼𝒩(1,0.1)$ and $\sigma_{i}∼𝒩(0.05,0.01)$. Within a single model, ρ is kept constant.

We chose the distributions for $\mu_{i}$ and $\sigma_{i}$ such that there could be variation in the distributions of $\Gamma_{i}$, while also maintaining values close to 1. If the values of Γ are very large or very small, Γ will overwhelm. W in the effective neural connectivity $W⊙\Gamma_{i}$. In other words, our assumed modulation does not re-scale synaptic weights by large amounts.

It should be noted that while we used vectors to construct our $\Gamma_{i}$ matrices, our modeling methodology will work with $\Gamma_{i}$ matrices of higher rank. Higher ranks of $\Gamma_{i}$, however, widen the parameter search space and constrain the optimization problem less. An analysis of HM-MINDy’s performance on synthetic data with $\Gamma_{i}$ matrices of rank 2 has been included in Appendix C, Fig. C.11.

To generate state changes in our synthetic data, we initialized a hidden Markov model (HMM) with transition probability matrix A and initial state probability π. At each timestep, we calculated the probability of the next state based on A and then randomly selected the state index weighted by the calculated probability. We chose A to be nearly diagonal, i.e,

(10) $$A=\left[\begin{array}{cccc} 0.9995 & 0.0005∕(m-1) & \cdots & 0.0005∕(m-1)\\ 0.0005∕(m-1) & 0.9995 & \cdots & 0.0005∕(m-1)\\ \vdots & \vdots & \ddots & \vdots \\ 0.0005∕(m-1) & 0.0005∕(m-1) & \cdots & 0.9995 \end{array}\right].$$

This enforces some degree of timescale separation between the dynamics of the neural activity and the dynamics of the modulation, as we are attempting to capture changes occurring on a much slower timescale than the neural activity. The initial state probability π was initialized uniformly, i.e.,

(11) $$\pi =\left[\begin{array}{c} 1∕m\\ \vdots \\ 1∕m \end{array}\right].$$

Once we had generated our random models, we forward simulated them for 20,000 timesteps (equivalent to 80 s of 250 Hz EEG) to create synthetic data. When fitting on this synthetic data, our models were initialized with the true lead field matrix and noise covariances (H, Q, and R). The other model parameters were initialized randomly and compared to the true values after fitting. Since the true value of W is known, however, we create a mask zeroing out the same entries in the fit W, to avoid zeroing out connections which are actually present in the synthetic models and artificially enforcing a worse parameter fit.

#### 2.3.2. EEG data

For our second experiment, we used EEG data of 20 subjects recorded with eyes open and eyes closed published in Cattan et al. (2018b) and available online (Cattan et al., 2018a). This data is highly compatible for our purposes because the different physiological regimes labeled in the data are expected to lead to distinctions in the posterior dominant rhythm (PDR) of EEG, and hence can be used as labels for validating our blind identification modeling paradigm.

**Fig. 2.**
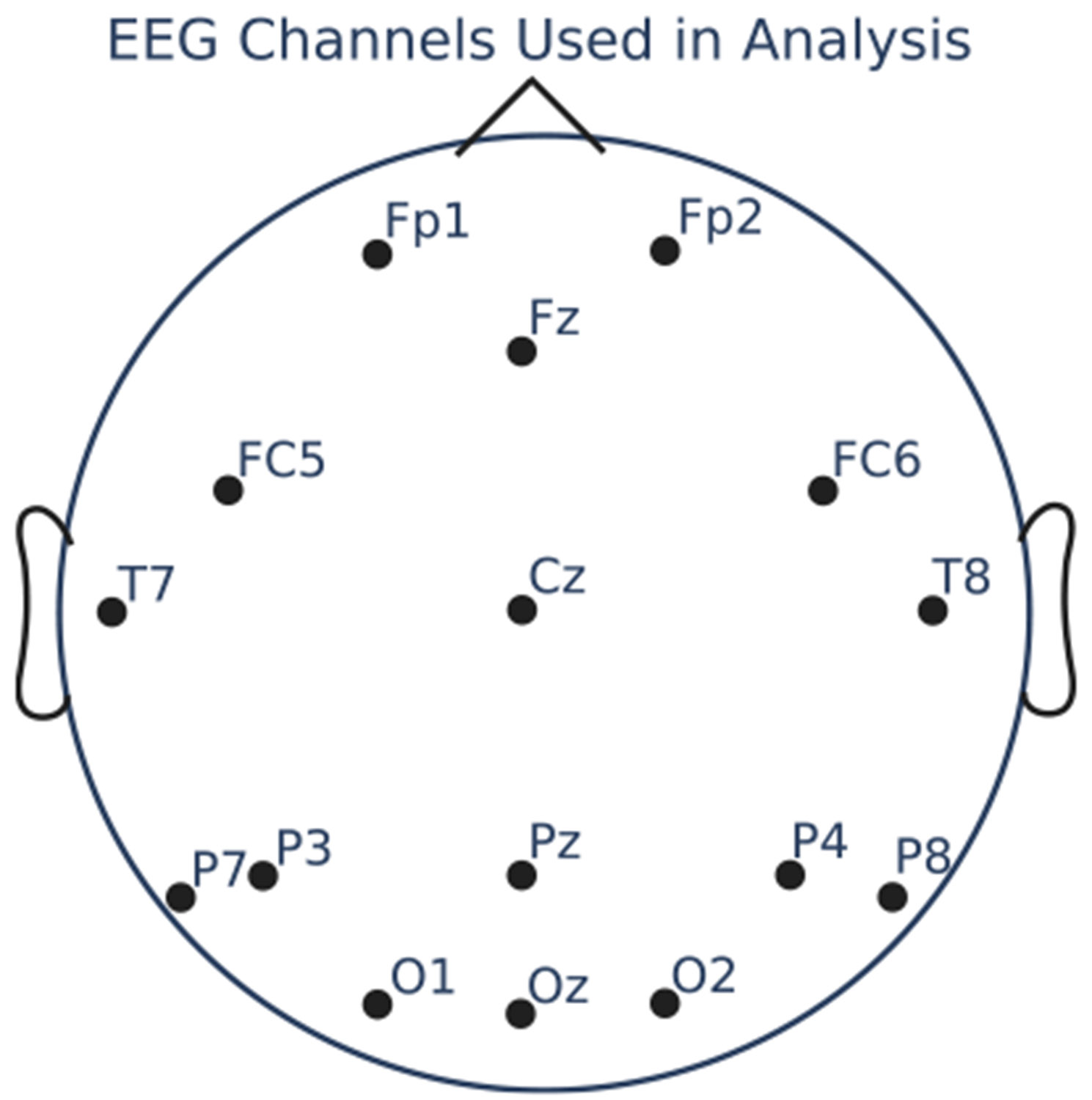
EEG channels used in analysis of eyes open/eyes closed EEG data.

We filtered the data between 8 and 12 Hz, subtracted the median of each channel, and divided by the mean absolute deviation of each channel. We added an eyes open/closed state index i∈{0,1} for each timepoint of the array, based on the events marking a switch in state included with the data. We used all 16 channels present in the data, shown in Fig. 2.

We performed multi-taper spectral analysis, obtaining spectrograms of each of the subjects as a baseline check for the presence of PDR alpha waves in the data, and confirmed that they were generally stronger in the eyes closed state, though the spectrograms were also quite variable throughout the cohort. Spectrograms of channel O2 for each of the 20 subjects can be seen in Appendix C, Fig. C.13.

#### 2.3.3. Forward model parameterization

Our H matrix was constructed as in (A.4):

(12) $$H=[H_{\text{exc}}\;\;0]$$

(13) $$H_{\text{exc}}=I-0.0511^{T},$$

where I denotes the 16 × 16 identity matrix and **1** denotes the 16-dimensional vector of all ones. We constructed R and Q as diagonal matrices, with R=1.2I and Q=0.25I. The other model parameters were initialized randomly, as in experiment 1. In this experiment, we do not have a ground truth of the 75% of non-diagonal connections in the $W_{\mathrm{ee}}$ and $W_{\mathrm{ei}}$ submatrices which are zero as we did in the synthetic data case. To continue enforcing this constraint, we selected a random 75% of non-diagonal connections in $W_{\mathrm{ee}}$ and $W_{\mathrm{ei}}$, and set these to 0 for all subjects.

## 3. Results

### 3.1. HM-MINDy is accurate and reliable

**Fig. 3.**
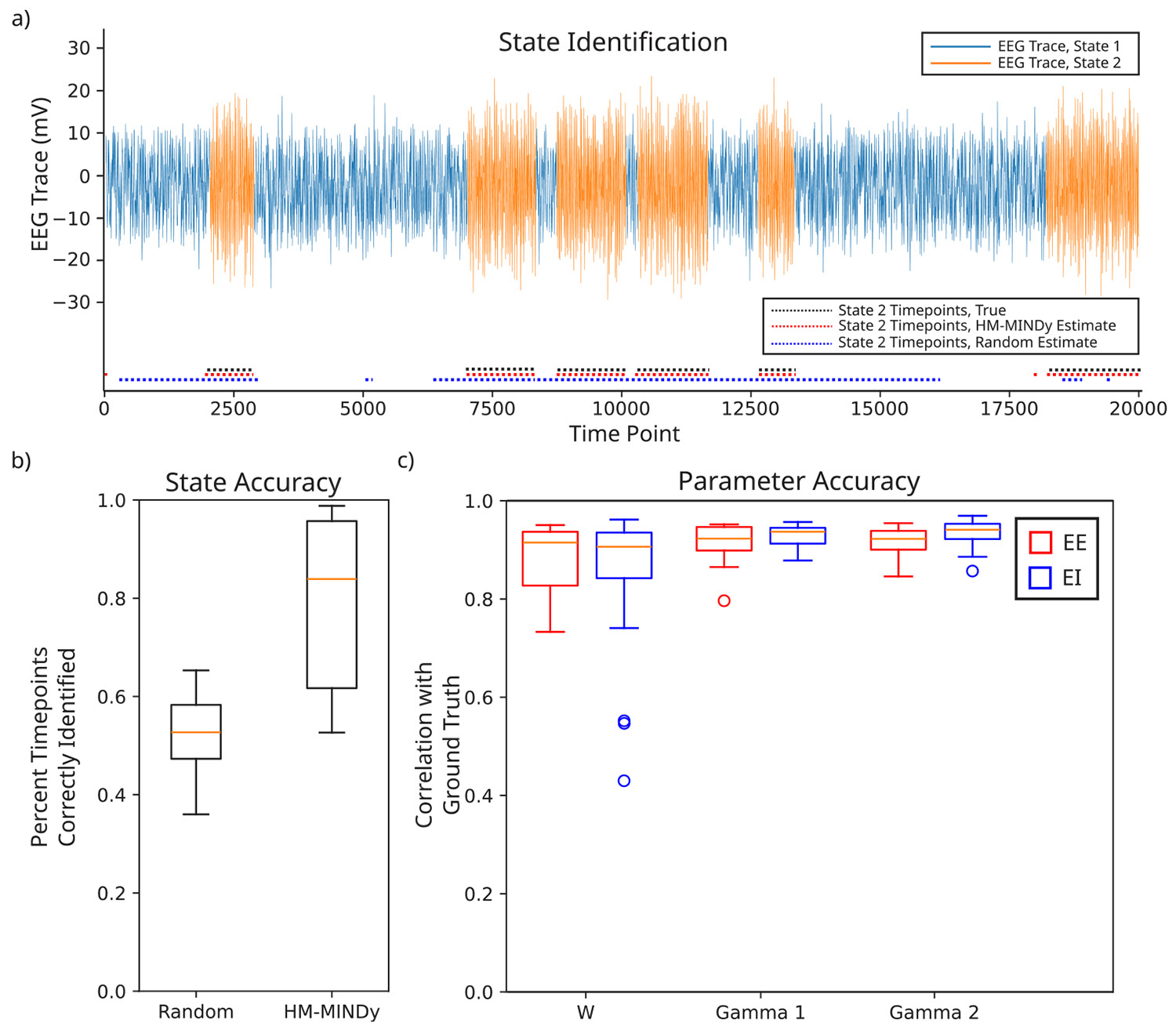
HM-MINDy accurately identifies both states and parameters in ground truth simulation data. (a) State identification of different latent states underlying an exemplar EEG channel. (b) State identification accuracy, defined as number of timepoints correctly identified for a random HMM and HM-MINDy. (c) Parameter accuracy (correlation with ground truth value) for EE and EI components of W, $\Gamma_{1}$, and $\Gamma_{2}$ matrices.

We first tested HM-MINDy on ground truth simulation data, in order to determine how well HM-MINDy can recover both known latent state changes, as well as known underlying dynamics. HM-MINDy significantly outperforms a random HMM with the same priors on the transition and initial state probability matrices (i.e., an HMM identical to the initialization values given in (10) and (11)). Often, the inferred HMM identified the latent states exactly or near-exactly (Fig. 3a). We found that on average, HM-MINDy correctly identified the latent HMM state in 84% of timepoints (IQR: 61%–96%), whereas a random HMM with the same priors correctly identified 52% of timepoints (IQR: 47%–59%) (Fig. 3b). The parameter accuracy was also high, with a correlation between the excitatory to excitatory (EE) components of the ground truth and fit W matrices of *r* = 0.92 (IQR: 0.82–0.94), a correlation between the EE components of the ground truth and fit $\Gamma_{1}$ matrices of *r* = 0.92 (IQR: 0.90–0.95), and a correlation between the EE components of the ground truth and fit $\Gamma_{2}$ matrices of *r* = 0. 92 (IQR: 0.90–0.95). The excitatory to inhibitory (EI) submatrices had similarly high correlations: W *r* = 0.91 (IQR: 0.83–0.94), $\Gamma_{1}$ *r* = 0.96 (IQR: 0.92–0.96), $\Gamma_{2}$ *r* = 0.95 (IQR: 0.92–0.96). These results are shown in Fig. 3c. When the zeroing mask is not applied, the state accuracy and the W correlation both remain high. These results have been included in Appendix C, Fig. C.12

### 3.2. HM-MINDy is robust

**Fig. 4.**
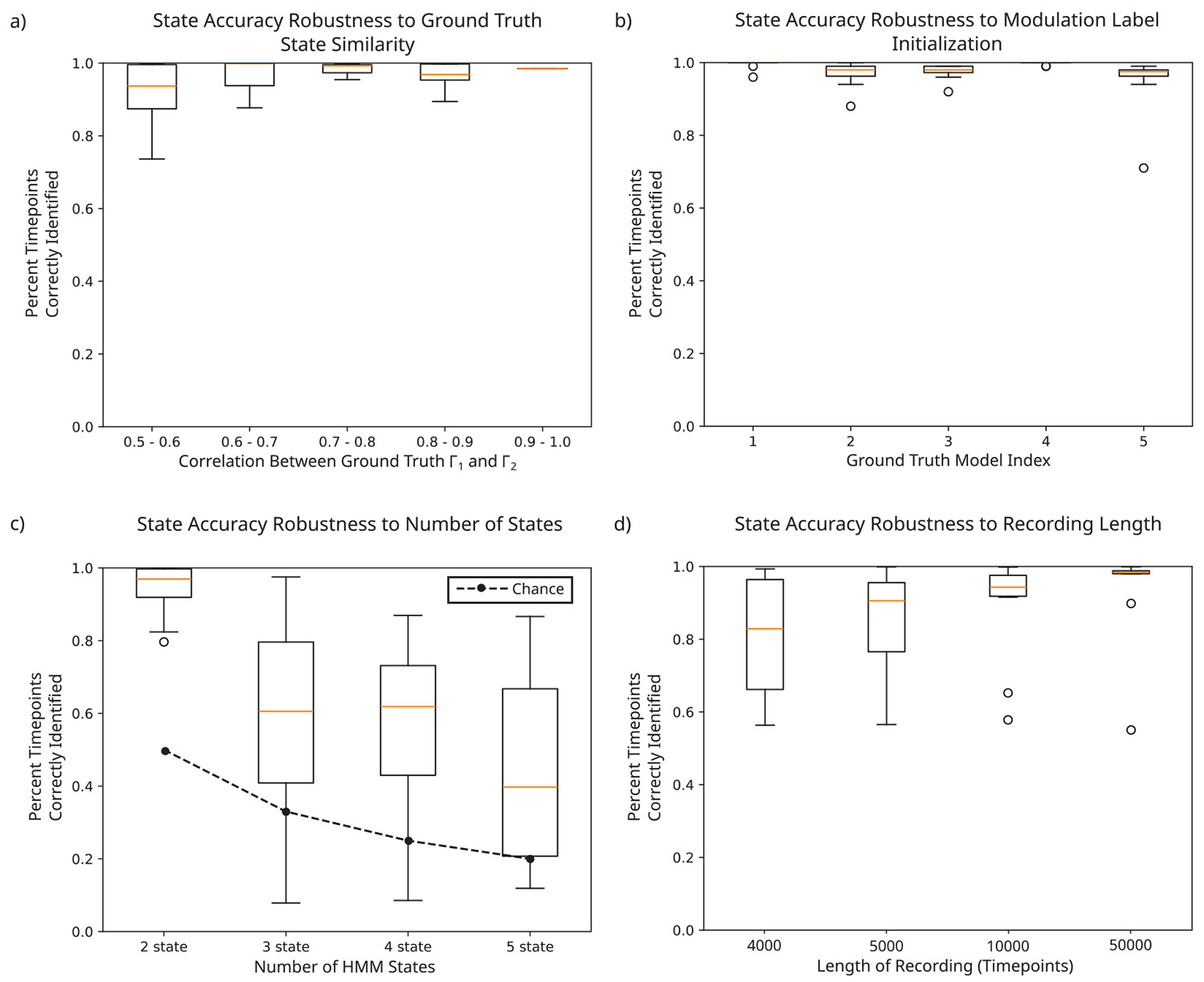
HM-MINDy is robust. (a) State identification accuracy compared to correlation between ground truth Γ matrices. (b) State identification accuracy distributions across different modulation index initializations for five synthetic data models. (c) State identification accuracy across different numbers of HMM states. (d) State identification accuracy across different lengths of synthetic data.

Next, we tested HM-MINDy’s robustness to varying levels of similarity between ground truth states. We found that HM-MINDy achieved over 90% state identification accuracy, regardless of the correlation coefficient of the ground truth Γ matrices (Fig. 4a).

We also tested HM-MINDy’s robustness to the initialization of the modulation labels. To test this, we fitted HM-MINDy models with ten different modulation label initializations to each of five sets of synthetic data and analyzed the state identification accuracy. We found that the state accuracy was consistently high across label initialization in all synthetic data sets, though there were occasional outliers with poor state accuracy. These results are summarized in Fig. 4b.

Additionally, we tested HM-MINDy’s robustness to the number of latent states embedded in the HMM. We simulated data with 2, 3, 4, and 5 true states and fitted an HM-MINDy model with the corresponding number of HMM states. We found that there was a drop in state identification accuracy with increasing numbers of latent states, but that the average state accuracy was significantly above chance in all cases (Fig. 4c).

Finally, we tested HM-MINDy’s robustness to the amount of data available, i.e., the length of the recording. To test this we created synthetic data ‘recordings’ ranging in length from 4000–50000 timepoints (equivalent to 16–200 s of 250 Hz EEG) and evaluated HM-MINDy’s ability to accurately detect the different states. We found that HM-MINDy was accurate down to recordings of around 5000 timepoints (20 s of 250 Hz EEG). Recordings shorter than that tended to have poor state identification accuracy. These results are highlighted in Fig. 4d.

### 3.3. HM-MINDy infers state differences from EEG recordings

**Fig. 5.**
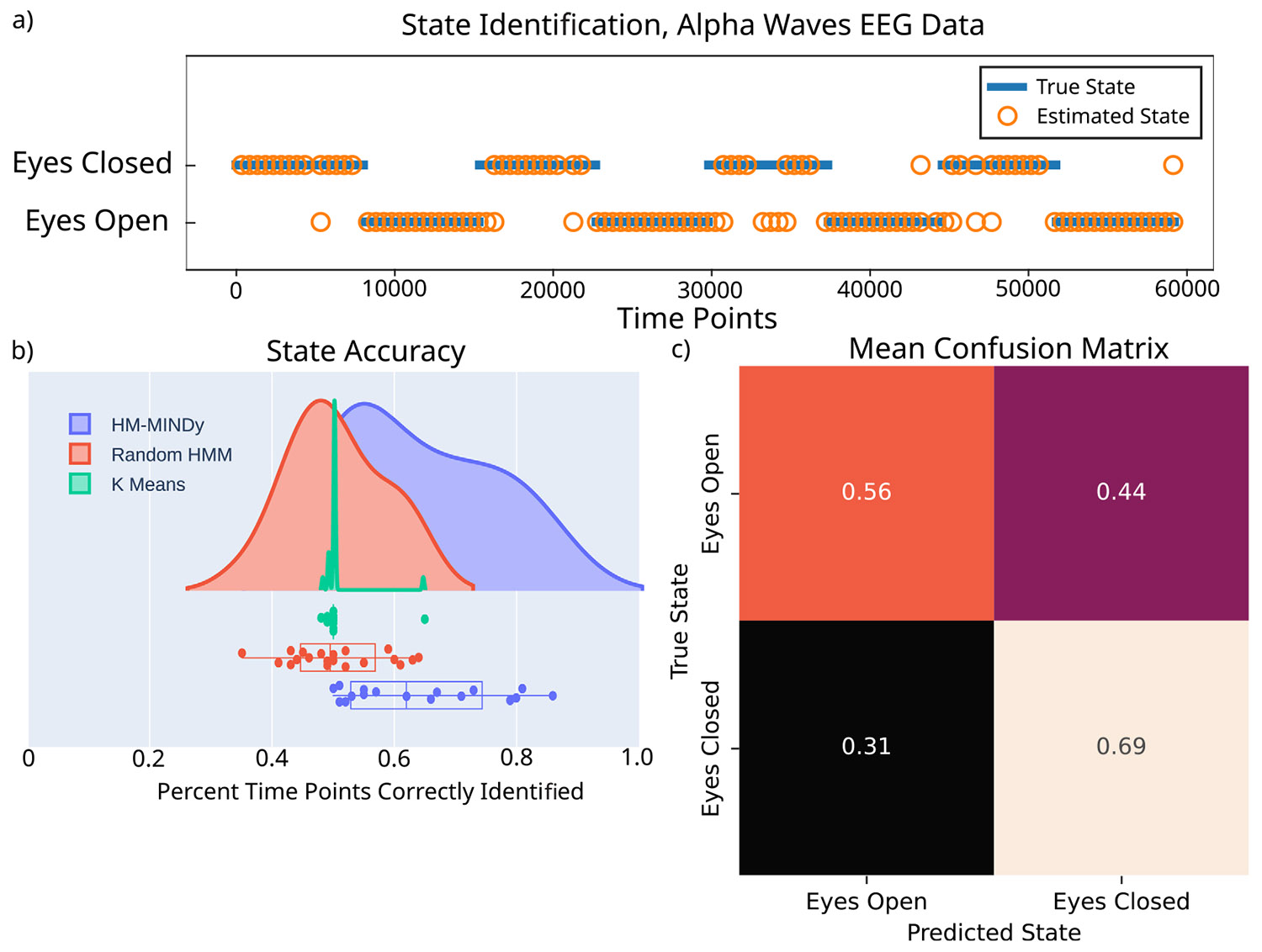
HM-MINDy infers state differences from EEG recordings. (a) State identification of different latent states underlying an exemplar EEG channel. (b) State identification accuracy, defined as number of timepoints correctly identified for HM-MINDy, a random HMM, and K-means clustering. (c) Mean confusion matrix across subjects, showing higher confusion for the eyes open state than the eyes closed state.

Finally, we tested HM-MINDy’s ability to infer state differences in EEG data. Specifically we used eyes open/eyes closed alpha wave data as a proof of concept for HM-MINDy’s state inference capabilities. We found that HM-MINDy significantly outperformed naive methods such as a random HMM and K-means clustering, achieving a mean of 61.5% of timepoints correctly identified compared to means of 50.45% (random HMM with the same priors) and 50.5% (K-means clustering), as shown in Fig. 5b.

In interpreting this result, we note the high variation of electrophysiological data between and within subjects, as shown in Fig. C.13. In some cases, the state identification is quite good (as in the exemplar in Fig. 5a). In other cases, the performance is less good with respect to the behavioral labels, but the method may nonetheless be correctly sensitive to variations underlying the actual EEG signal. For a more complete discussion of this, see 4.1.

We noted that the eyes closed state was more often well identified, while the eyes open state was less well identified. To quantify this, we created a confusion matrix of the number of time points correctly and incorrectly identified in each state. The mean confusion matrix is shown in Fig. 5c, and each subject’s is shown in Appendix C Fig. C.14. We hypothesize that this confusion in the eyes open state is due to the fact that the data has been filtered to the alpha-band frequency range (8–12 Hz), so any differences in signal content are in magnitude rather than frequency.

**Fig. 6.**
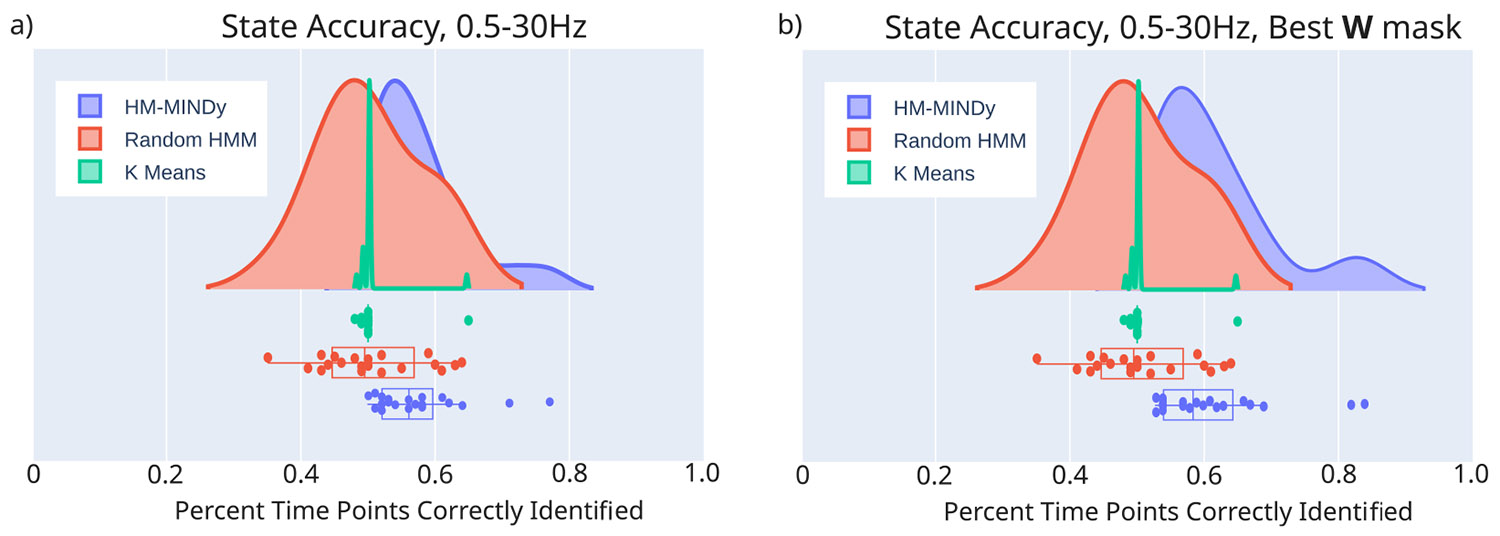
Effects of frequency band and W mask on state identification accuracy. (a) State identification accuracy, defined as number of timepoints correctly identified for HM-MINDy on data filtered between 0.5–30 Hz, a random HMM, and K-means clustering. (b) State identification accuracy, defined as number of timepoints correctly identified for HM-MINDy with the best: W mask on data filtered between 0.5–30 Hz, a random HMM, and K-means clustering.

We performed our modeling on EEG data which had been filtered to the alpha band (8–12 Hz) since we are attempting to detect an alpha-band phenomenon. Nevertheless, it is important to understand how HM-MINDy performs when a broader frequency band is utilized. For this reason we repeated our EEG modeling on the eyes open/eyes closed data, filtered between 0.5–30 Hz. We found that in this case the performance suffered a decrease in median accuracy of 6 percentage points, decreasing from 62% (IQR: 53%–75%) to 56% (IQR: 52%–58%), though this still outperforms both K means and random segmentation (Fig. 6a). We hypothesize that this decrease in performance may be due to HM-MINDy attempting to model a more complex signal with the same number of parameters as was used to model the simpler, more aggressively filtered signal.

To mitigate this, we turned to the mask used on our connectivity matrix W. We initially used a random W mask across all subjects, which may not be well-tuned spatially to the connections which most effectively model the data. To customize the connectivity mask, one could use structural connectivity data from an individual to determine the connections most likely to be non-zero, as was done by Singh et al. for (unmodulated) MINDy (Singh et al., 2025). Since structural connectivity is not available for this dataset (and indeed, is often unavailable in clinical settings), we performed a Monte-Carlo analysis, fitting an HM-MINDy model with 5 different random W masks, and choosing the one which yielded the highest state accuracy. This raised our performance back up to similar levels as the single random mask on the more aggressively filtered data, yielding a median state accuracy of 59% (IQR: 54%–66%), shown in Fig. 6b. This Monte-Carlo method of determining the best mask then improves the accuracy of the model, but comes at the cost of significantly increasing the time necessary to fit the models. Therefore, it is necessary to consider the tradeoffs of both filtering the data and masking the connectivity matrix when using HM-MINDy for modeling work.

### 3.4. HM-MINDy accurately captures frequency domain content from time domain signals

**Fig. 7.**
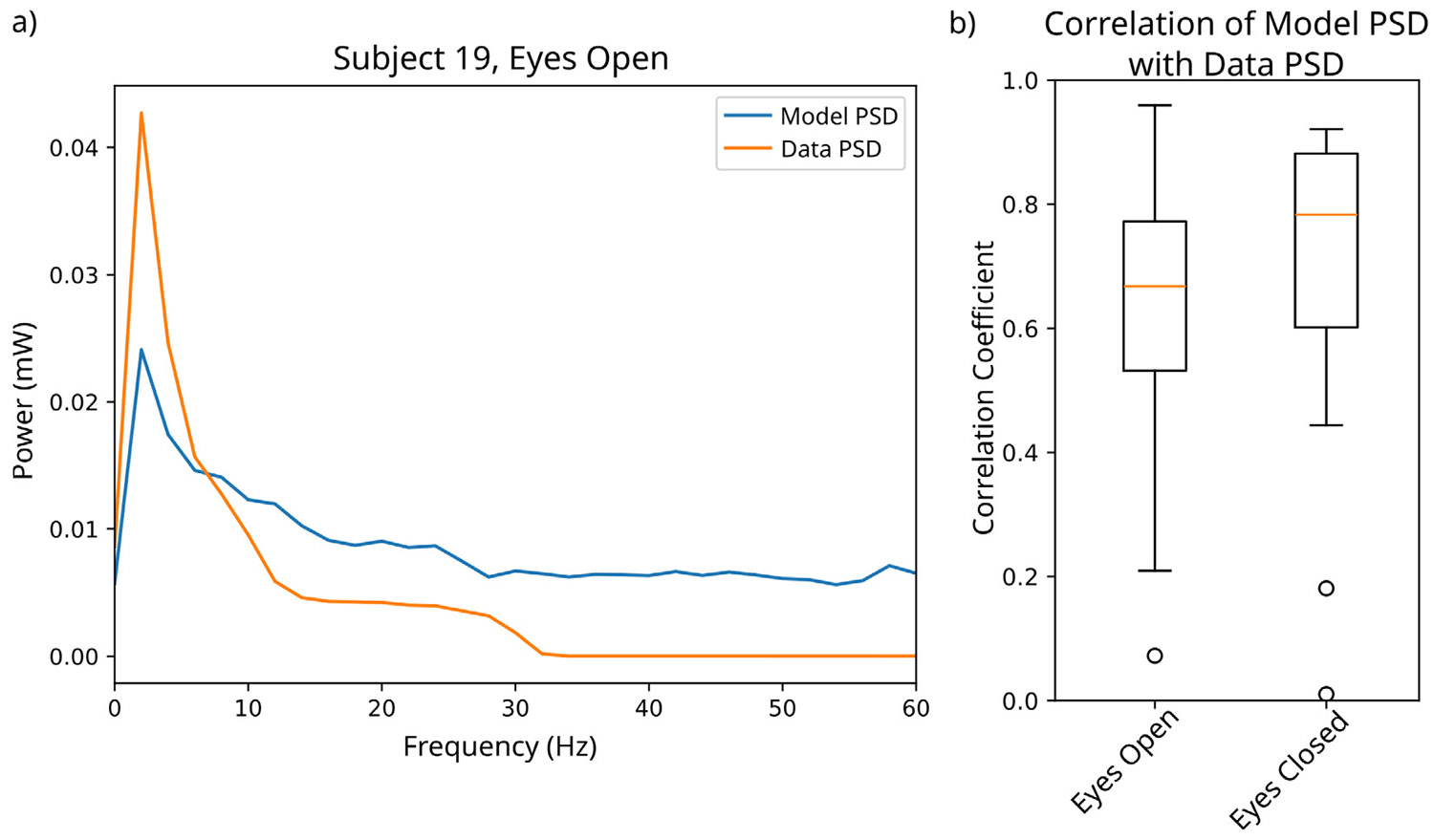
HM-MINDy accurately captures frequency domain content from time domain signals. (a) Exemplar power spectral density estimates from data generated by the model (blue) and actual data (orange). (b) Correlation of power spectral density estimates for eyes open and eyes closed states.

While MINDy and modulated MINDy (i.e., the inner loop of HM-MINDy) have been demonstrated to provide accurate and interpretable results on EEG data (Schwamb et al., 2024, 2025), we also tested the accuracy of the models inferred by HM-MINDy. To test this, we compared the frequency structure of the actual data to the frequency content of data generated from the fit models. We found that in both the eyes open and eyes closed states, the power spectral density estimate of the data generated by the model typically correlated well with the power spectral density estimate of the actual data. In the eyes open state, we observed a median correlation of 69% (IQR: 57%–79%), and in the eyes closed state we observed a median correlation of 76% (IQR: 59%–86%), as shown in Fig. 7. This indicates that the models fitted in HM-MINDy not only lend themselves to accurately capturing the changes in latent state, but also capture the dynamics embedded in the EEG data.

### 3.5. HM-MINDy infers hidden Markov models which generalize to unseen data

**Fig. 8.**
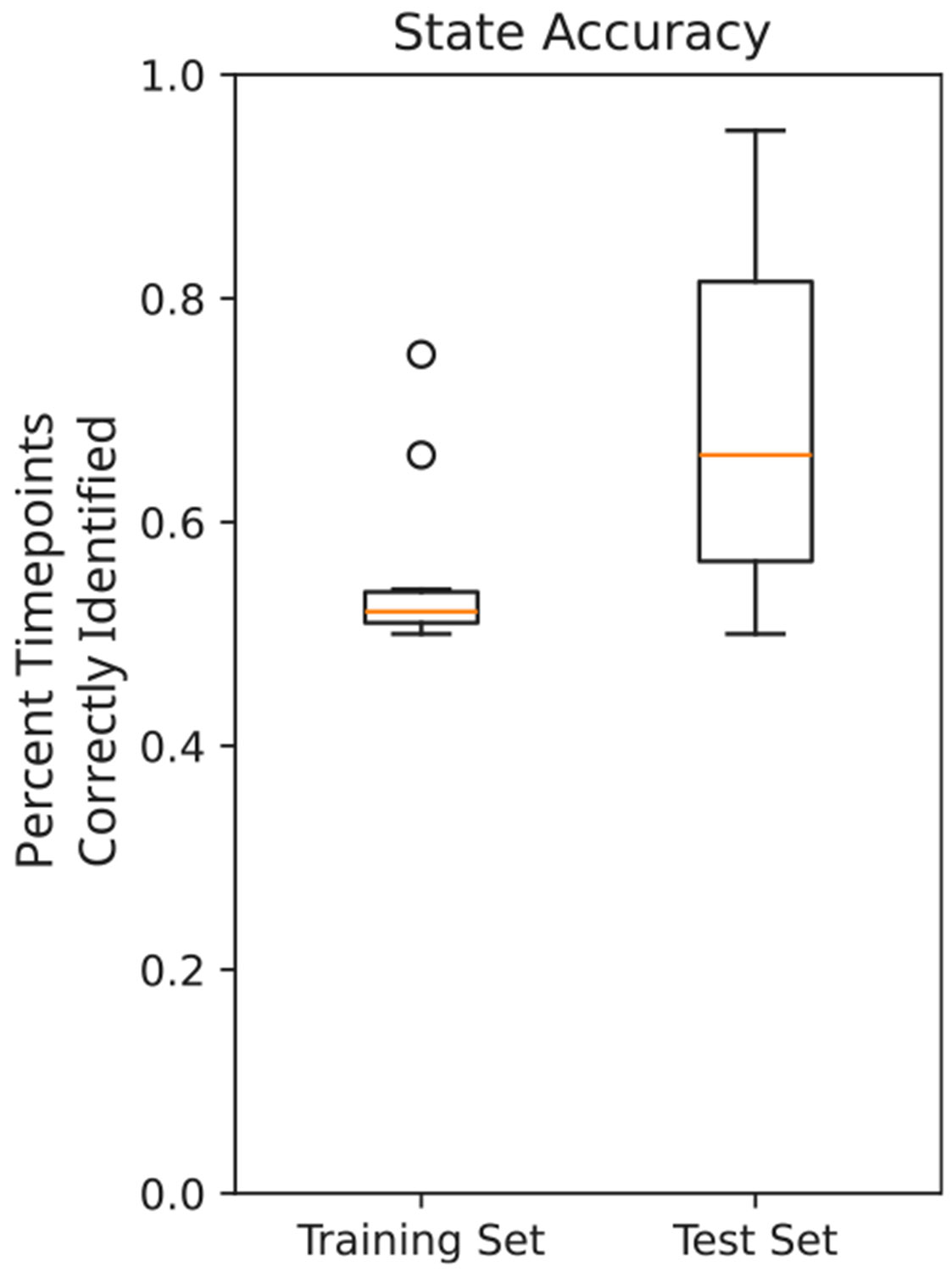
State identification accuracy on training and test data.

While labeling and modeling observed data is the crux of our problem, it is also informative to know how generalizable the generated models are. MINDy and modulated MINDy have been shown to be reliable across time in test-retest analyses (Singh et al., 2020, 2025; Schwamb et al., 2025), but the dynamics of the inferred Hidden Markov Models underlying the modulatory states have not yet been tested for generalizability to unseen data. To test this, we fit HM-MINDy models on the first 75% of each recording, holding out the last 25% as a test set. We then held both the MINDy model and HMM parameters constant and tested state identification on the last 25% of the recordings. We found that the state identification accuracy on this test set was comparable or higher than the state identification accuracy on the training set (Fig. 8). This indicates that the inferred HMMs do accurately capture the underlying dynamics of the transitions in neural dynamics, and can potentially predict when it is likely that a subject will undergo a change in modulatory state.

### 3.6. HM-MINDy eliminates unnecessary states

**Fig. 9.**
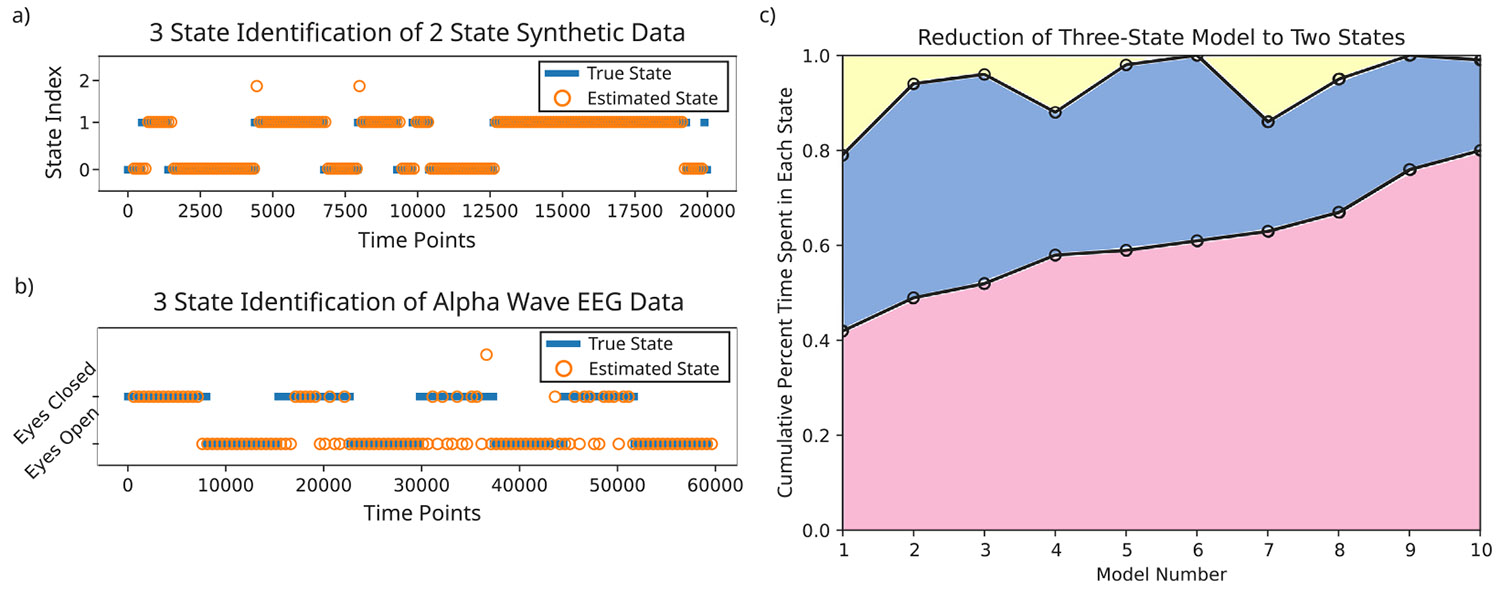
HM-MINDy eliminates unnecessary states. (a) State identification when three states were specified for the HMM on synthetic data containing only two distinct states. (b) State identification when three states were specified for the HMM on the eyes open/eyes closed EEG data. (c) Cumulative percentage of time points identified in each state in three-state models fit on two-state synthetic data.

Notably, HM-MINDy requires that the user pre-specify the number of states that HM-MINDy is expected to infer from the data. In cases such as the eyes open/eyes closed data used in this paper, a fairly reasonable guess can be made that HM-MINDy should infer two states. In other cases, however, the choice of the number of states may not be quite as clear. To understand how important it is to accurately specify the number of states present in the data, we specified that HM-MINDy fit three states on both two-state synthetic data as well as the eyes open/eyes closed alpha wave data. In these cases, HM-MINDy reduced the number of identified states to essentially two states, spending minimal time in the third state (Fig. 9). Thus, when the number of states or dynamical regimes in the data are unknown, it is possible to estimate an approximate number of states and get a good fit, erring on the side of an excess of specified states.

## 4. Discussion

### 4.1. HM-MINDy is sensitive to variation in EEG signal

**Fig. 10.**
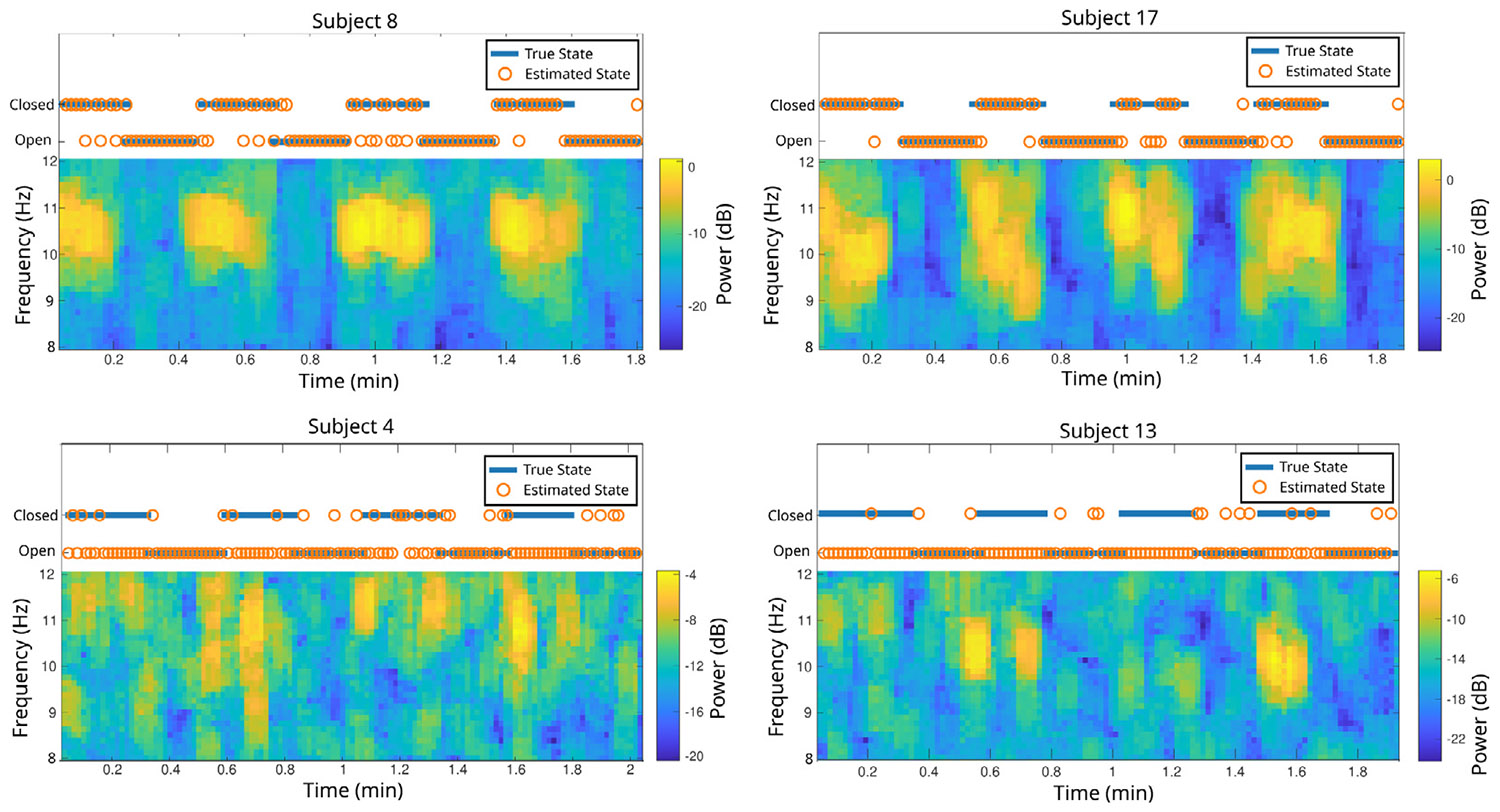
HM-MINDy is sensitive to variation in EEG signal. Spectrograms and identified states for two subjects with particularly good performance (top) and particularly bad performance (bottom).

While all subjects were recorded with eyes open and eyes closed for similar lengths of time, there is significant variation in the spectral content of the EEG recordings, both within and between subjects. This variation would imply variability in the underlying circuit dynamics, leading to discordant alignment between inferred changes in dynamical regime, vs. labeled behavioral regime. For example, subjects 8 and 17 have quite well-defined and consistent spectral differences between the eyes open and eyes closed states. Subjects 4 and 13, on the other hand, have less clearly defined spectral differences. These differences are highlighted in Fig. 10, and spectrograms for all subjects can be seen in Fig. C.13. When comparing the state identification performance for the four subjects highlighted here, there is a clear difference between the state identification performance on subjects 8 and 17 (80% and 86% of time points correctly identified, respectively), and the performance on subjects 4 and 13 (50% and 53% identified correctly), also shown in Fig. 10. Thus, it seems evident that HM-MINDy is describing changes in *dynamics inferred from data*, as is our goal. That these changes do not always align with behavior could arise for many reasons, from simple data quality, to more complex individual variation in activity-function relationships. Ultimately, the purpose of HM-MINDy is to enable an assessment of such questions by enabling the segregation of the data on the basis of identified dynamics.

### 4.2. HM-MINDy enables point-wise inference of both state changes and latent dynamics

As noted in the introduction, there are a lack of methods that provide the ability to infer both changes in dynamical regime evident in non-stationary data and the latent dynamics of each regime. The approach developed in this paper represents an expansion of current direct-parameterization techniques for modeling brain dynamics, specifically an expansion upon our mesoscopic individualized neural dynamics (MINDy) framework. By expanding MINDy to model data with multiple dynamic regimes present, we enable detection and modeling of changes in data that may or may not be evident by eye. We anticipate that this will have particular impacts in analysis of clinical data, where labeled changes in neural dynamics may be limited or lacking.

A key feature of HM-MINDy is that it operates in a point-wise fashion. That is, the inference is done at every time-point, in the time-domain. This is in contrast to potential alternatives in the frequency domain, wherein some amount of windowing of the data is needed in order to obtain spectral estimates (i.e., as in the spectrograms of Fig. 10). Such spectral methods would also be inherently sensitive to various choices in spectral estimation itself.

### 4.3. HM-MINDy enables parsing of constant and state-dependent dynamics

A key aspect of HM-MINDy is that it decomposes the connectivity into a non-stationary, switched modulation of a constant weight matrix, an architecture we previously introduced in Schwamb et al. (2025). This enables some level of continuity across the switched dynamic regimes, reflecting the fact that though varying over time, brain dynamics do not switch to completely independent dynamical regimes. Moreover, this approach enables inference of both constant connectivity dynamics across time (the unmodulated model, without Γ), as well as dynamics specific to each dynamic regime.

As mentioned in the introduction, there is some commonality between our formulation and Li et al. (2024), which tackles a similar problem of identifying non-stationarity with modulation as an assumption. However, there are important distinctions. At the highest level, the modeling paradigms used are distinct (i.e., statistical vs. dynamical formulations, targeting different spatial scales of description and data modalities). For this reason, it is not appropriate nor is it technically feasible to compare or benchmark these methods without fundamentally altering or re-developing one or the other. That said, there are also common elements to note. Li et al. also decompose a connectivity matrix into the Hadamard product of two component matrices, but with the different intent. Li et al. construct their connectivity matrices as the product of a one-hot matrix representing the direction of connectivity, and a positive matrix representing the strength of the connection. To enforce continuity between dynamical regimes, Li et al. impose a Gumbel-Softmax similarity penalty on the one-hot matrices, and a Gaussian similarity penalty on the positive matrices. By contrast, we encode the direction of connectivity into our model by specifying excitatory and inhibitory populations (and thus connections) *a priori*, and do not allow these to change over time. For more details of the specific directed structure of the connectivity matrix W, see Appendix A. Since the direction of the connections is so deeply embedded in the model, both our W and Γ components encode information about the strength of the connections, and Γ can be viewed as modulating only the *strength* of the base W connections, and not the direction of connectivity. Therefore, our model encourages continuity between dynamical regimes, and parses out the stationary and non-stationary aspects of brain dynamics.

### 4.4. Limitations

A number of limitations are present in this study. First, as discussed above, HM-MINDy requires the user specify an estimated number of distinct states/dynamical regimes. Though this specification is robust to a specified number of states larger than the true number of states, it is not robust to a smaller number of specified states. Second, HM-MINDy models *discrete* changes in dynamic regime — one and only one dynamic regime is ‘active’ at any time. The dynamics are unified by our constant base weight matrix W, but it is not clear that dynamic regimes switch in such a discrete manner within the brain. To fully accomplish non-stationary modeling of neural dynamics, a continuously varying system may be necessary. Finally, HM-MINDy performs less well on the measured EEG data than the synthetic data. More experiments and analysis are needed to understand why this is the case and what steps can be taken to mitigate this. Nevertheless, HM-MINDy significantly outperforms naive solutions for segmenting non-stationary data, while also inferring multiple sets of dynamics. Additionally, as discussed in 4.1, HM-MINDy captures changes in dynamics underlying electrophysiological data, which may align imperfectly with labels based on behavioral changes.

As alluded to in 4.3, direct comparison of HM-MINDy with other state-of-the-art methodology is not feasible. While the SRNNs of Karniol-Tambour et al. (2024) or Zhang and Saxena (2024), as well as the approach of Li et al. (2024), are solving problems closely aligned to our problem, the specifics of these models are highly distinct from the specifics of HM-MINDy. Karniol-Tambour et al. Zhang & Saxena, and Li et al. all use spike data, which operates at a different spatial scale of recording than M/EEG. This leads to Karniol-Tambour et al. and Zhang & Saxena to implement models which *reduce* the dimensionality of the neural data to a low-dimensional manifold, whereas we are *inferring* the neural activity of hidden populations. Additionally, our modulation architecture inherently introduces a higher-dimensional parameter space by virtue of including one matrix per modulation state ($\Gamma_{i}$) as well as the base connectivity matrix (W). Karniol-Tambour et al. and Zhang & Saxena both only utilize one connectivity matrix per modulation state, and thus have a smaller number of parameters to fit. These differences in spatial scale, dimensionality reduction/expansion, and connectivity matrix architecture make it difficult to compare the methods of Karniol-Tambour et al. Zhang & Saxena, and Li et al. directly to HM-MINDy on their own terms. Adaptations to account for these differences may be possible, but would introduce differences that would make interpretation of the comparison tenuous.

### 4.5. Conclusion

In conclusion, we have presented HM-MINDy, a methodology for blindly identifying latent changes in dynamics along with inferring the dynamics themselves. We have validated this approach on synthetic ground truth data and shown its robustness to both the similarity of the ground truth states as well as the number of latent dynamic regimes. We also showed a proof of concept application on eyes open/eyes closed data. In the future, we hope to improve HM-MINDy’s performance on noisy data, and update the inference procedure to run in real-time, rather than merely retrospectively on data which has already been gathered. We anticipate that HM-MINDy’s ability to describe dynamics of non-stationary brain data will be useful for many clinical and scientific applications.

## Supplementary Material

1

## Figures and Tables

**Table 1:** Parameter values or random distributions used to create synthetic data models. **I** denotes the identity matrix, and **1** denotes the vector of all ones.

Parameter	Description	Initialization value
$W_{s}$	Sparse part of connectivity matrix	$(16∕20)𝒰{\left(0,1\right)}^{3}$
$W_{l1}$, $W_{l2}$	Low rank part of connectivity matrix	$𝒰{\left(0,1\right)}^{3}+0.2𝒰(0.1)$
$S_{\mathrm{exc}}$	Excitatory nonlinearity slope	2.5
$S_{\mathrm{inh}}$	Inhibitory nonlinearity slope	1
V	Nonlinearity offset	0
$D_{\mathrm{exc}}$	Excitatory decay	0.65+0.02𝒰(0,1)
$D_{\mathrm{inh}}$	Inhibitory decay	0.8+0.02𝒰(0,1)
C	Baseline neural activity	0
$H_{\mathrm{exc}}$	Lead field for excitatory populations	𝒩(0,1)
Q	Measurement noise covariance	0.25I
R	Process noise covariance	(0.2+0.1𝒰(0,1))I
nS	Number of modulation states	2
$\mu_{i}$	Mean of $\Gamma_{i,k}$	𝒩(0,0.1)
$\sigma_{i}$ (uniform)	Variance of $\Gamma_{i,k}$	𝒩(0.4,0.1)
$\sigma_{i}$ (normal)	Variance of $\Gamma_{i,k}$	𝒩(0.05,0.01)
$\Gamma_{i,k}$ (uniform)	Vector constructing $\Gamma_{i}$	$𝒰(\mu_{i}-0.5\sigma_{i}$, $\mu_{i}+0.5\sigma_{i})$
$\Gamma_{i,k}$ (normal)	Vector constructing $\Gamma_{i}$	$𝒩(\mu_{i},\sigma_{i})$
A	HMM transition probability	$0.9995I+(0.0005∕nS)(11^{T}-I)$
π	Initial HMM state probability	(1∕nS)1

## Data Availability

The data is already available at the location cited in the text, url: https://doi.org/10.5281/zenodo.2605110.

## Appendix A. Model formulation

The base (i.e., unmodulated) dynamics of the MINDy model are governed by:

(A.1)
$$x_{t+1}-x_{t}=W\;\tanh(\mathrm{Sx}+V)+\mathrm{Dx}+C+\epsilon_{t}$$

(A.2)
$$y_{t}={\mathrm{Hx}}_{t}+\nu_{t},$$

Where $x_{t}\in R^{n}$ represents the neural activity of n neural populations at time t, and {W,S,V,D,C} are the tunable model parameters. Here, W is the connectivity matrix, D is a diagonal matrix representing the decay (leak) in neural activity for each population, S and V parameterize the slope and offset of the sigmoidal activation function, and C represents a baseline bias of each neural population. H is the lead field model which translates the population-level neural activity into the measured data, and $\epsilon_{t}$ and $\nu_{t}$ represent the process noise and measurement noise, respectively.

Note that this model is mathematically comparable to a vanilla recurrent neural network, but arranged and constrained to reflect biologically interpretable relationships between excitatory and inhibitory neural populations. By assigning neural populations to spatial locations in the brain and constraining the parameters to specific valence (i.e., positive/excitatory, negative/inhibitory), we can examine changes in these parameters associated with physiological changes. Specifically, we enforce excitatory and inhibitory substructures onto the neural population activity vector $x_{t}$ and the connectivity matrix W like so:

(A.3)
$$x_{t}=\left[\begin{array}{c} x_{t,\text{exc}}\\ x_{t,\text{inh}} \end{array}\right],\;W=\left[\begin{array}{cc} W_{\text{ee}} & W_{\text{ie}}\\ W_{\text{ei}} & W_{\text{ii}} \end{array}\right].$$

Entries in $W_{\mathrm{ee}}$ and $W_{\mathrm{ei}}$ are constrained to be positive or 0, as they represent the connections from excitatory neural populations. Additionally, $W_{\mathrm{ee}}$ and $W_{\mathrm{ei}}$ are full submatrices. Conversely, $W_{\mathrm{ie}}$ and $W_{\mathrm{ii}}$ have entries which are negative or 0, as they represent the connections from inhibitory neural populations. Since inhibitory neurons only have local connections (Kätzel et al., 2011), these submatrices are constrained to be diagonal.

The specification of H is made to reflect the specifics of the data modality being used for model construction. In our case, because we are using cortical data (EEG), we account for prevailing hypotheses regarding the contributions of different cell types to surface-level potentials. In this context, it is generally believed that inhibitory neurons are not close enough to the surface of the cortex, nor do they possess the spatial organization, to generate fields detectable via EEG (Buzsáki et al., 2012). Therefore, we construct our lead field matrix H to have zeros in the submatrix multiplied by the inhibitory component of x:

(A.4)
$$H=[H_{\text{exc}}\;\;0]\;.$$

We adapted and generalized this model by adding neuromodulation via matrices $\Gamma_{i},i=1,\ldots ,m$ multiplied element-wise by the connectivity matrix W:

(A.5)
$$x_{t+1}-x_{t}=(W\;⊙\;\Gamma_{i})\tanh(\mathrm{Sx}+V)+\mathrm{Dx}+C+\epsilon_{t}.$$

Here, m represents the number of modulatory states to be modeled. This modulation structure is schematized in Fig. 1d. To preserve the signed connectivity structure of W, entries in each $\Gamma_{i}$ matrix are constrained to be positive or 0. This enables a modulation which scales entries in W by varying amounts, without affecting the base structure of the connectivity.

## Appendix B. Backpropagated Kalman filter approach

To address the dual estimation basis expansion problem, we take an iterative approach combining an Extended Kalman Filter (Kalman, 1960; Julier and Uhlmann, 1997) with backpropagation of error gradients. First we apply a Kalman filter to a small window of data in order to estimate the state. We then evolve the model forward from the (estimated) state to generate a forward prediction. We then backpropagate the error through both the free simulation steps and the Kalman filtering steps, calculating the error gradient for each parameter at each step. In addition to calculating the parameter error gradients (i.e., error gradients of {W,Γ,D,C,S,V}), we also calculate the error gradients of the estimated covariances of the noise terms ϵ and ν. These noise covariances are necessary for the Kalman filter to estimate the current state $x_{t}$, and are optimized alongside the model parameters. By backpropagating the error in this manner and fitting both the parameters and the noise covariances, we fit a model which produces the most accurate state estimator which can best predict future measurements.

After backpropagation through the free simulation and Kalman filtering steps, we then update the model parameters and the estimates of the noise covariances. Then, the process is repeated with a new small window in the epoch of fitting data. This process of Kalman filtering, free simulation and backpropagation is repeated with new data windows until the Kalman and free simulation errors converge. Multiple windows of the data epoch are used so that the model captures the general dynamics and statistical properties of the entire epoch, to avoid overfitting to a few timesteps of data. These windows are selected at random to avoid biasing the fit toward any one period within the epoch (e.g., the beginning or end of the epoch).

## Appendix C. Supplementary data

Supplementary material related to this article can be found online at https://doi.org/10.1016/j.jneumeth.2025.110600.
