# Supplementary material for: Blind identification of state transitions and latent neural dynamics from electrophysiological recordings
Source: J Neurosci Methods. Author manuscript; Available in PMC 2026 Apr 30. (PMC13131968; doi:10.1016/j.jneumeth.2025.110600)
Supplement: 1 [file NIHMS2163576-supplement-1.pdf]

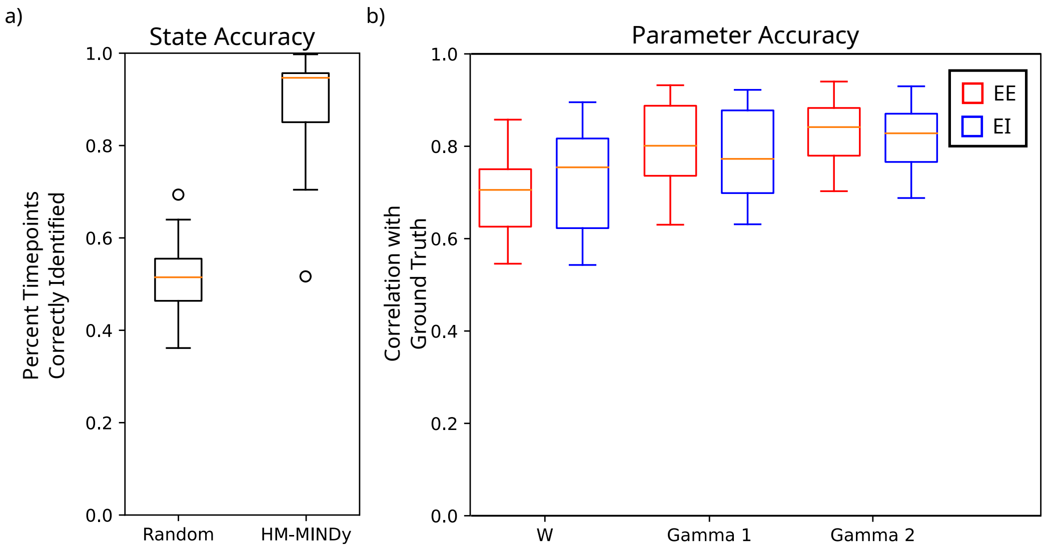

Figure C.11: **State and parameter accuracy with rank 2 Γ_i.**

Appendix C. Supplementary Figures

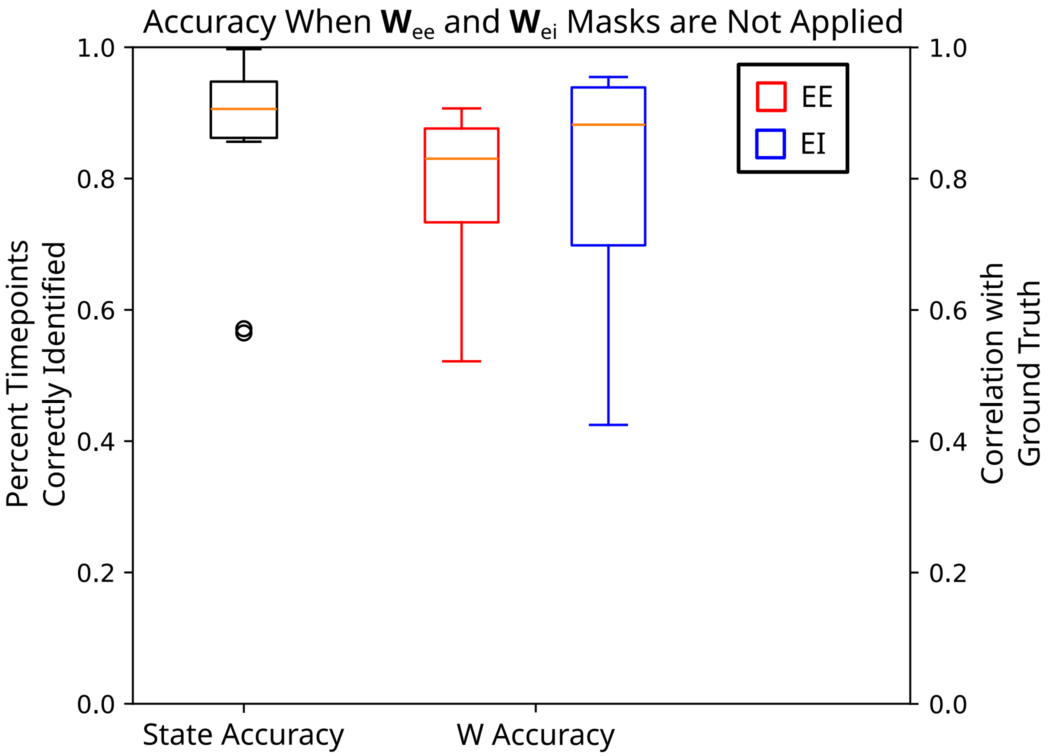

Figure C.12: **State and W accuracy with no mask imposed on excitatory connections.**

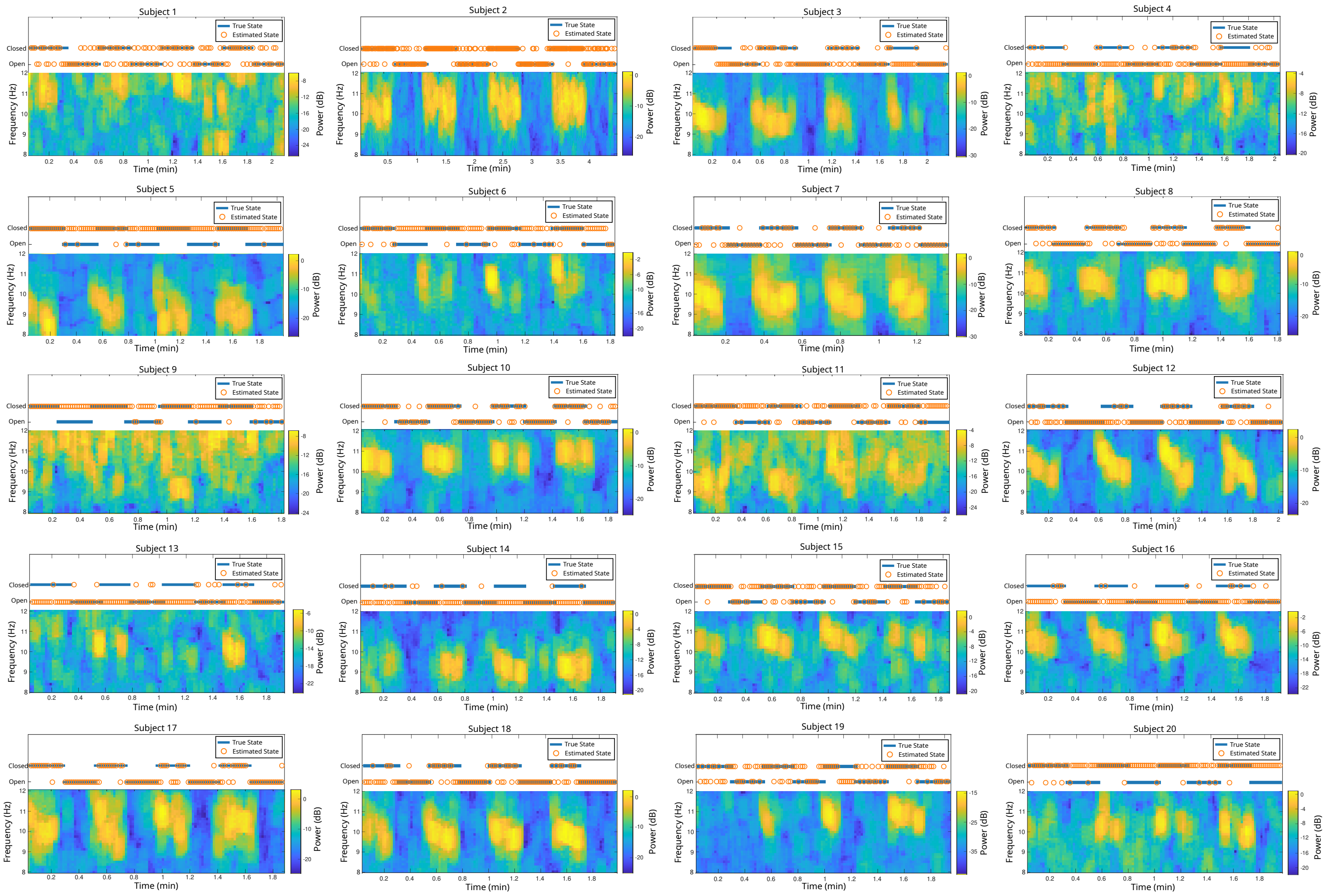

Figure C.13: **Individual subject spectrograms and state identification results**

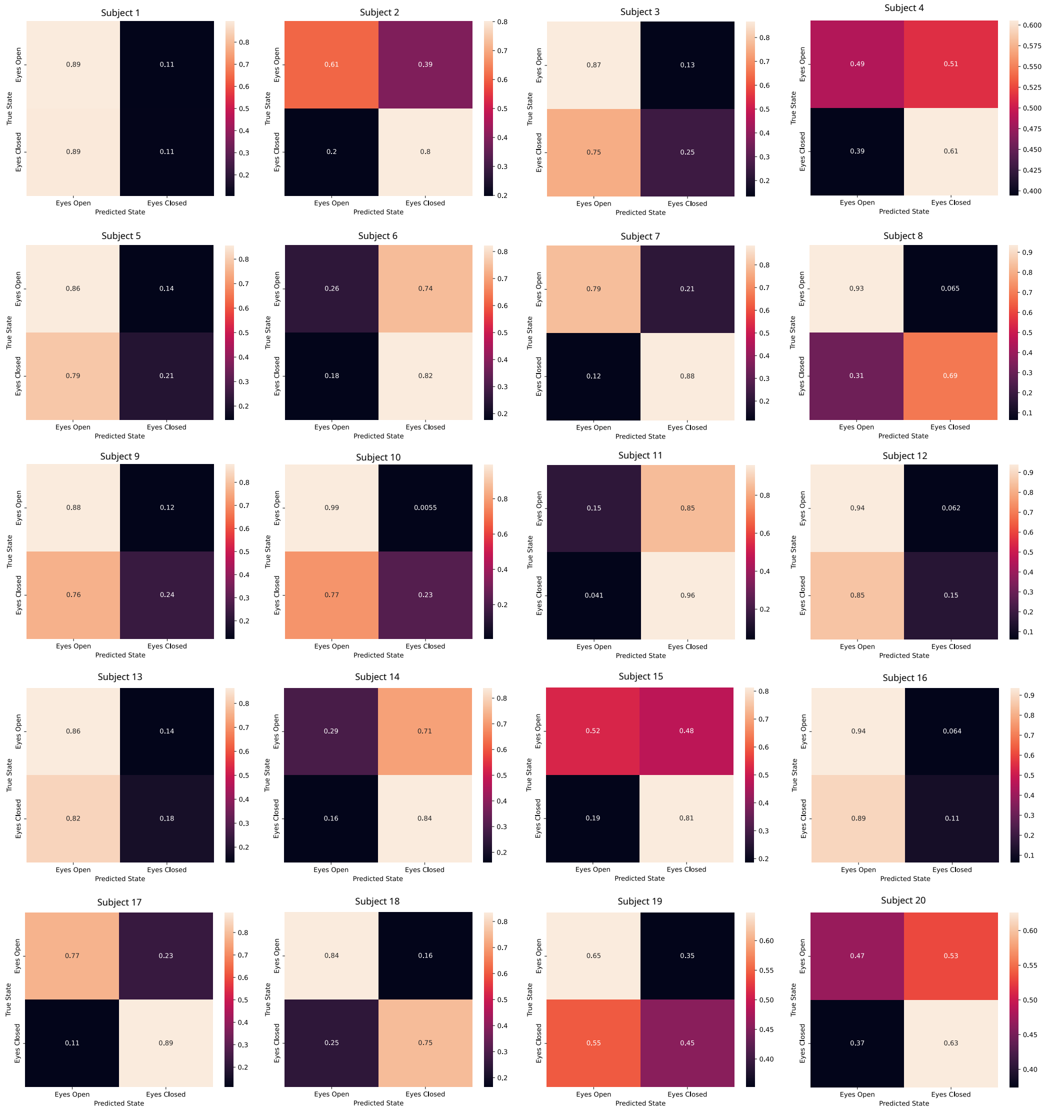

Figure C.14: **Individual subject confusion matrices**
